# Assessment of disease control in allergic rhinitis

**DOI:** 10.1186/2045-7022-3-7

**Published:** 2013-02-18

**Authors:** Pascal Demoly, Moises A Calderon, Thomas Casale, Glenis Scadding, Isabella Annesi-Maesano, Jean-Jacques Braun, Bertrand Delaisi, Thierry Haddad, Olivier Malard, Florence Trébuchon, Elie Serrano

**Affiliations:** 1 Allergy Division, Pulmonary Department, INSERM U657-EA2415, Hôpital Arnaud de Villeneuve, University Hospital of Montpellier, Montpellier, France; 2 Section of Allergy and Clinical Immunology, Imperial College London-NHLI, Royal Brompton Hospital, London, UK; 3 Division of Allergy and Immunology, Department of Medicine, Creighton University, Omaha, NE, USA; 4 Rhinology Department, Royal National Throat, Nose and Ear Institute, London, UK; 5 INSERM U707 and Faculté de Médecine Pierre et Marie Curie, Paris, France; 6 Hôpital de Hautepierre and Nouvel Hôpital Civil, Strasbourg, France; 7 Hôpital Robert Debré, Paris, France; 8 Hôpital Tenon, Paris, France; 9 Centre Hospitalier Universitaire de Nantes, Nantes, France; 10 Private office, St Clement de Rivière, France; 11 Hôpital Larrey, Toulouse, France

**Keywords:** Allergic rhinitis, Disease control, Disease severity, Classification, Questionnaire, ARIA, GINA

## Abstract

The Allergic Rhinitis and its Impact on Asthma (ARIA) initiative has had a significant impact, by raising awareness of allergic rhinitis (AR) and improving the diagnosis and treatment of AR sufferers. ARIA classifies the severity of AR as "mild" or "moderate/severe" on the basis of "yes"/"no" answers to four questions. This two-point classification has been criticized as providing little guidance on patient management; patients with "mild" AR are unlikely to consult a physician, whereas the group of patients with "moderate/severe" seen by specialists is heterogeneous. These perceived shortcomings have prompted attempts to improve the ARIA classification or, by analogy with the Global Initiative for Asthma (GINA), adopt approaches based on "disease control" in AR. Even though "disease severity", "disease control" and "responsiveness to treatment" are different (albeit related) metrics, they are not mutually exclusive. Currently, there is no single, accepted definition, but we propose that "disease control" in AR can combine (i) measurements of the severity and/or frequency of daily or nocturnal symptoms, (ii) impairments in social, physical, professional and educational activities, (iii) respiratory function monitoring and (iv) exacerbations (e.g. unscheduled medical consultations and rescue medication use). Although control-based classifications have a number of limitations (e.g. their dependence on treatment compliance and the patient's psychological status), these instruments could be used as an adjunct to the ARIA severity classification and regional practice parameters. Here, we assess the strengths and weaknesses of the current two-level ARIA classification, analyze published proposals for its modification and review the literature on instruments that measure AR control. We conclude that there is a need for research in which severity is compared with control in terms of their effects on patient management.

## 

Allergic rhinitis (AR) is a highly prevalent, chronic disease, with rates of up to 50% in some populations [1,2]. Furthermore, the prevalence is increasing in many "westernized" countries [3]. Its disease burden is considerable - with negative impacts on sleep, mood, social functioning, work/school performance and health-related quality of life [4-6] and can no longer be neglected by healthcare payers since this burden is associated with direct health resource costs and indirect socio-economic costs (e.g. absenteeism and loss of productivity) [7]. Furthermore, the detrimental effects of AR on established asthma and the link between AR and the subsequent development of asthma are well established [8]. Paradoxically, this disease burden tends to be underestimated by both patients and physicians [9]. In Europe, over half of AR sufferers do not seek medical advice [10]. Treated patients also report poor levels of satisfaction, with a constant search for a combination of medications that "works" by reducing their nasal symptoms [11].

Challenged by these patient needs and the lack of awareness among patients and physicians, a number of global, regional and local initiatives and guidelines for improving the diagnosis, treatment and follow-up of AR sufferers have been developed [12]. One of the key initiatives relates to the output of the 1999 Allergic Rhinitis and its Impact on Asthma (ARIA) workshop (organized by the World Health Organization), published in 2001 and updated in 2008 and 2010 [13-15]. To aid the implementation of a stepwise approach to patient management, ARIA introduced a patient classification based on the AR symptoms' time patterns ("intermittent" vs. "persistent") and severity ("mild" vs. "moderate/severe"). Although the ARIA severity classification has been validated in primary care patients [2,16], surveys have found that both primary care physicians (PCPs) and specialists are not necessarily aware of this tool [17]. Lastly, regulatory authorities tend to consider that AR cannot truly be severe and/or uncontrollable.

By analogy with trends in the management of asthma following the introduction of the Global Initiative for Asthma (GINA) guidelines [18]), there is a general, WHO-endorsed trend towards the generalization of the "control" approach to other conditions, including AR, chronic rhinosinusitis, chronic urticaria and atopic dermatitis [19]. As discussed below, there is no single definition of "disease control", since the variables taken into account and the severity thresholds corresponding to "relief" vary from one tool to another. However, by analogy with GINA in asthma, measurements of "control" in AR can combine (i) measurements of daily or nocturnal symptoms, (ii) impairments in social, physical, professional or educational activities, (iii) respiratory function monitoring and (iv) events related to exacerbations (such as medical consultations and the need for rescue medication). Hence, long-term, stable disease control equates to minimal symptoms, no limitations in activities, minimal use of rescue medications and infrequent exacerbations.

The objective of the present work was to (i) assess the strengths and weaknesses of the ARIA classification in terms of guiding the treatment of AR, (ii) review published proposals for the modification of ARIA and (iii) review instruments for determining disease control in AR. We searched MEDLINE, Embase and the Cochrane Library up until May 2012 using logical combinations of the following terms (in English only): allerg*; rhinit*; ARIA; "Allergic Rhinitis and its Impact on Asthma"; control; questionnaire; rating; scale; score.

## Strengths and weaknesses of the current ARIA severity classification

The ARIA classification was clearly a great step forward in 2001, since it acknowledged the impact of a disease that was often qualified as “trivial" and emphasized the need to assess patient needs and treat accordingly. The ARIA "mild" vs. "moderate/severe" classification [13-15] has a number of strengths and weaknesses (Table 1). It has the advantage of being very simple to administer, since it is based on "yes"/"no" answers to each of the following statements: "My symptoms disturb my sleep", "My symptoms restrict my daily activities (sports, leisure, etc.)", "My symptoms restrict my participation in school or work" and "My symptoms are troublesome". In ARIA, "mild" AR corresponds to "no" answers to all four questions, whereas the presence of a "yes" for one or more items corresponds to "moderate/severe" AR. The ARIA duration and severity classifications have been implemented in several countries and patient populations. For example, the ADRIAL and PEDRIAL cohort studies of adult and paediatric AR patients in Spain [20,21] found that symptom, Rhinitis Quality of Life Questionnaire (RQLQ) and visual analogue scale (VAS) scores were significantly higher in "moderate/severe" than in "mild" AR. In France, Bousquet et al. studied 3052 patients consulting PCPs and who were classified according to the ARIA classification (mild intermittent: 11%; mild persistent: 8%; moderate/severe intermittent: 35%; moderate/severe persistent: 46%). All patients were scored for the RQLQ, the Jenkins sleep questionnaire and the Allergy-Specific Work Productivity and Activity Impairment questionnaire [22]. Impairments were correlated more strongly with severity than duration. Eighty per cent of the patients with moderate-to-severe AR reported impaired activities, as opposed to only 40% of the patients classified as having mild AR.

**Table 1:** Strengths and weaknesses of the two-level ARIA severity classification ("mild" vs. "moderate/severe") for AR

**Strengths**	**Weaknesses**
• Easy to apply	• Some duplication between questions
• Patient-centred	• "Mild" patients unlikely to seek treatment
• Emphasizes the existence of severe allergic rhinitis	• "Moderate-severe" patients form a heterogeneous group
• Correlated with disease-specific quality of life, sleep quality, work productivity and visual analogue scale scores	• Poor uptake by physicians (both primary care physicians and specialists)
	• Not extensively applied by physicians - even those who are aware of the classification
	• Does not take account of past and present treatments

Levels of awareness and application of the ARIA severity classification are less than satisfactory. In a study of 943 French PCPs and 277 ear, nose and throat (ENT) specialists, Demoly et al. found that only about 54% of the physicians were aware of the ARIA classification. Over 90% of these actually applied the classification in practice - although there were significant differences between PCPs and ENT specialists [17]. Furthermore, knowledge of the ARIA classification by PCPs did not appear to influence the use of H1-antihistamines (H1As) and/or intranasal corticosteroids (ICSs) as a function of the patient's disease severity [17]. In another cohort study performed in France [23], researchers found that ARIA severity did not significantly influence medication prescription. Although ARIA suggests that patients with mild and intermittent AR should receive H1As and those with moderate/severe and persistent AR should receive ICSs, Ramirez et al.'s analysis of 3026 patients with intermittent AR and 3507 patients with persistent AR (enrolled by 1346 practitioners) revealed that immediate prescription of H1A+ICS combination therapy was surprisingly frequent [23].

For AR patients seen by specialists, the "mild" vs. "moderate/severe" distinction has less value since by definition, a patient with "mild" AR is not bothered by his/her symptoms and is unlikely to consult a specialist. In Valovirta et al.'s survey of patients aged 16 and over [9], 87% of those with persistent conditions and 79% of those with intermittent conditions reported that at least one daily activity was moderately or severely affected “all the time”. Similarly, Van Hoecke et al. observed that 89.3% of patients consulting PCPs in Belgium were classified as "moderate-severe" [24], as was the case for 92.2% of the patients in the above-mentioned French cohort [17,25].

However, “moderate-severe” AR is extremely broad (with as few as one or all four ARIA items impaired) and encompasses a highly heterogeneous group of patients. It includes patients with severe chronic upper airway disease (SCUAD) [26], candidates for allergen specific immunotherapy or nasal obstruction surgery and patients with severe disease in the absence of treatment but who respond well to ICSs or even H1As alone. As such, classification of a patient as suffering from “moderate-severe” AR is of little help in guiding the physician's treatment recommendations. In this respect, it has even been suggested that term "moderate/severe" should be replaced by "severe" alone [22]. Lastly, the ARIA classification does not take account of past and present treatment; this is an important shortcoming, since most AR patients seen for the first time by a physician will have already taken prescription and/or over-the-counter medications for their condition. In summary, consideration of the ARIA classification's particular features has highlighted unmet needs in the routine clinical management of AR.

## Attempts to refine, improve or simplify the ARIA classification

Since the publication of ARIA's initial work in 2001, several attempts have been made to refine the "mild" vs. "moderate/severe" classification. In 2007, Valero et al. suggested drawing a distinction between 1 to 3 affected ARIA items on one hand and 4 affected items on the other, with the assignment of "moderate" and "severe" disease grades to these respective situations [27]. In a study of over a thousand treatment-naïve patients, classification as "mild", "moderate" or "severe" was correlated with the disease-specific "Cuestionario ESPañol de Calidad de Vida en RINiTis" (ESPRINT-15) quality of life score [28].

In a study in Belgium, Van Hoecke et al. suggested the use of just two ARIA questions (one question on sleep disturbance and one on impairment in daily life) and the introduction of an additional, "moderate" category [24]. Two "no" replies equated to "mild" AR, one "yes" and one "no" equated to "moderate" AR and two "yes" replies equated to "severe" AR [24]. However, in a cohort of 5140 patients in France classified as having mild (n=357, 7.0%), moderate (n=2498, 48.6%) or severe AR (n=2285, 44.4%) according to Van Hoecke et al.'s suggestion, Demoly et al. found that "no clear clinically relevant trends were observed that could support the need for a distinction between mild, moderate and severe patients" [29]. In response to these proposals to classify AR severity as “mild”, “moderate” and “severe”, the ARIA 2008 authors stated that this "makes it more complex for the practising physician without bringing significant improvement to the patient since this more complex classification does not translate to a difference in therapeutic options" [14].

## Visual analogue scales

Visual analogue scales have been suggested as simple tools for assessing AR severity. Firstly, Bousquet et al. [30] found that a 0 to 10 cm VAS score and the RQLQ score were significantly correlated (rho = 0.46; p <0.0001) and that a patient with a VAS score below 5 cm could be classified as having "mild" AR (negative predictive value: 93.5%), whereas a VAS score over 6 cm was equated with "moderate/severe" AR (positive predictive value: 73.6%). Indeed, the ARIA 2008 guidelines subsequently included a classification in which "mild" AR = 0–3 cm, "moderate" AR = 3.1–7 cm and "severe" AR = 7.1–10 cm. Secondly, the United States' Joint Task Force on Practice Parameters [31] suggested that six VASs (sneezing, runny nose, congestion, itchy nose, postnasal drip and total nasal symptoms) could be used. However, the Task Force's 2003 publication did not present any results on application of these VASs and the validation status of this instrument is uncertain. Thirdly, two of the present authors (PD and IAM) and colleagues compared a VAS severity score with a numerical severity score (both assessed by a physician) in a sample of 36,000 patients with diagnosed, non-complicated, untreated, intermittent AR [32]. Although the two scores were correlated, the absolute values differed and 23.86% of the patients were classified as "severe" according to one scale but not the other.

## Instruments for assessing disease control in allergic rhinitis

Disease control is now being considered as an alternative to disease severity in the management of patients with respiratory disease. Indeed, the assessment of disease control in AR was briefly mentioned in the ARIA 2008 update [14], since "as for asthma, one of the problems to consider is to replace severity by control". However, the ARIA authors noted that, at the time the update was drafted, "sufficient data are not yet available" and that "control questionnaires or methods are still undergoing validation" [14]. Since then, however, a number of AR control questionnaires have been built and validated according to the ideal methodological sequence [33]. These questionnaires are summarized in Table 2.

**Table 2:** A comparison of three published allergic rhinitis control questionnaires

	**CARAT **[33-35]	**RCAT **[36,37]	**ARCT **[38]
*Administration mode*	self-questionnaire	self-questionnaire	self-questionnaire
*Diseases considered*	allergic rhinitis and asthma	allergic rhinitis	allergic rhinitis
*Period of evaluation*	The previous 4 weeks	The previous week	The previous 2 weeks
*Number of final items/questions*	17 in development, 10 in the final tool	26 in development, 6 in the final tool	5 in the final tool
*Response type*	4-point frequency scale and some yes/no items	5-point Likert scale	5-point frequency scale
*Validation status*	Tested in 141 non-treated adult patients (CARAT17) and then 193 adults (CARAT10). Internal consistency over 0.70. Longitudinal validation in 51 patients at 4 outpatient clinics. Test-retest reliability (intra-class correlation coefficient) = 0.82	Psychometric validation by 410 patients consulting allergy specialists. God psychometric properties and reliable internal consistency (Cronbach alpha coefficient: 0.70)	Tested in 902 patients selected by 411 primary care physicians and allergists. Internal consistency: 0.77
*Other comments*	Tested in patients consulting an allergist	Significant correlations with physician-rated disease severity, total nasal symptom score and physician-recommended change in therapy	Based on the Asthma Control Questionnaire. Significant correlations with the clinical picture and the impact of allergic rhinitis on social and sports activities

The Control of Allergic Rhinitis and Asthma Test (CARAT) was initially developed by Nogueira-Silva et al. in Portuguese [34]. After a literature search, Nogueira-Silva et al. included 17 questions in a questionnaire with a Likert scale. The reference period was four weeks – long enough to monitor control over time but short enough to be unaffected by recall bias. A ten-question version of CARAT (CARAT10) was subsequently validated in a cross-sectional study of 193 adults by Fonseca et al. [35]. The range of possible scores for CARAT10 is 0–30, 0 being the complete absence of control. The CARAT10 served as a guide to patient management, since the mean [95% confidence interval] scores associated with the intensification, maintenance and reduction of treatment were 15 [13.6-16.5], 21 [19.4-21.9] and 24 [21.4-26.6], respectively.]. Most recently, Fonseca et al. investigated the CARAT in 62 patients included at 4 outpatient clinics in Portugal [36]. At two visits 4 to 6 weeks apart, a total of 51 patients (aged between 18 and 70) completed the CARAT10, the Asthma Control Questionnaire [37], three VASs (on airways symptoms, bronchial/pulmonary symptoms and nasal symptoms) and an overall self-assessment of control. Lung function was also tested. The test-retest reliability (intra-class correlation coefficient) was 0.82. A significant change in the CARAT10 score was observed in clinically unstable patients. In terms of the change over time in the CARAT10 score, the correlation ranged from 0.49–0.65 for the ACQ5 and VAS symptom scores and from 0.31 to 0.41 for the physician's assessment of control [36].

Nathan et al. developed a 26-item Rhinitis Control Assessment Test (RCAT) and refined it to 6 items. After testing the RCAT in 410 AR patients [38,39], six of the 26 initial items (nasal congestion, sneezing, and watery eyes, sleep interference, activity avoidance and self-assessed control) were most predictive (p <0.001 for all) of the allergist’s overall rating of rhinitis symptom control.

By working with a multidisciplinary group associating allergists, pulmonologists, ENT physicians and methodologists, Demoly et al. developed a five-item, self-assessment Allergic Rhinitis Control Test (ARCT) [40] with similarities to the Asthma Control Test (ACT) [41]. Two ACT questions (on rescue medication and overall assessment of the disease) were incorporated into the ARCT. The questionnaire was validated by testing in 902 patients (selected by 411 PCPs and allergists) before treatment and two weeks after treatment. The score at inclusion correlated significantly (p<0.0001) with the patient's overall clinical status and the impact of AR on social and sporting activities. A significant (p<0.0001) increase in the score was observed after two weeks of treatment (from 14.9±4.0 at inclusion to 21.5±2.9 after treatment). Using a receiver operating characteristic curve, a score of 20 was found to be the optimal cut-off for poor vs. well-controlled rhinitis (sensitivity: 67%; specificity: 82%; negative predictive value: 32%; positive predictive value: 95%).

The "Allergy-Control-SCORE"^TM^[42] measures (i) the severity of 10 nasal and non-nasal symptoms on a 4-point scale) and (ii) medication use (out of a catalogue of 745 different medications) and combines the two metrics with equal weighting. In a study of 81 patients with allergic rhinoconjunctivitis and 40 healthy controls, the Allergy-Control-SCORE was significantly correlated with the global assessment of allergy severity, the RQLQ score and the number of medical consultations due to allergy within the previous year. In our opinion, "Allergy-Control-SCORE" is something of a misnomer because this approach is conventionally referred to as a "combined score" (i.e. a combination of a symptom score and a medication score) and has been extensively used in clinical trials for many years [43].

Lastly, Scadding et al. took a different approach by asking experts to complete an online survey about what they regarded as rhinitis control in relation to the seven questions in Juniper's mini-RQLQ [44]. All the respondents considered that more than “somewhat troubled” was unacceptable. Indeed, most of the experts defined control as being “hardly troubled at all” by each symptom.

Unfortunately, head-to-head comparisons of these various control-based tools have not been reported.

## Discussion

### Disease severity vs. disease control

Although "disease severity", "disease control" and "responsiveness to treatment" are different (albeit related) metrics, they are not mutually exclusive [45]. Disease severity can be defined as a loss of physiological function caused by the disease process. Both severity and control can be measured in a multitude of ways, with both objective and subjective measurements and patient-reported vs. physician-reported outcomes. Patient-reported metrics are growing in importance in clinical research and, increasingly, in patient care [46], although there is debate over whether the physician or the patient is best placed to judge disease control [47]. There is a need for research in which severity is compared with control in terms of the respective effects on patient management.

Conceivably, some cases of severe disease might respond well to treatment (i.e. good control), whereas some cases of mild disease might not (i.e. poor control). Likewise, a totally controlled patient taking an H1A and an ICS will probably still have severe underlying disease [19]. Furthermore, poor disease control may be related to poor treatment compliance and psychosocial factors rather than high disease activity. Mild disease may be a problem for some patients but not for others. Conversely, severe disease may bother some patients far less than others.

Severity can be measured in treatment-naïve patients but, by definition, the concept of disease control is only applicable in treated patients. Hence, conventional measurements of severity will continue to be essential in treatment-naïve patients consulting for the first time or in undiagnosed AR sufferers in the general population. In this respect, the VAS appears to be a valid shortcut for the definition of disease severity. However, a one-dimensional scale cannot encompass the complex spectrum of parameters involved in disease control (i.e. impairments in everyday life, respiratory function and exacerbations, in addition to symptom severity).

Control-based classifications have a number of limitations. Some are related to the underlying concept of measuring disease control over the previous weeks, since factors such as treatment compliance and the patient's psychological status will have an effect on perceived disease control – even when intrinsic disease activity is constant. It is not yet clear whether AR control varies significantly as a function of the disease-inducing allergen. For example, it is possible that the achievement of control in patients with persistent AR induced by house dust mites is very different from that in patients with AR induced by grass pollen. This is a complex area requiring further research.

Lastly, the questionnaires used to evaluate AR control have been developed and validated in adolescents and adults. However, by analogy with tools such as the Childhood Asthma Control Test [48], extension to children with AR can be envisaged.

### The potential impact of disease control instruments on clinical practice

The ARIA guidelines state that "treatment should be tailored according to the severity of the disease, comorbidities, treatment availability and affordability and patients’ preference". Two recent papers co-authored by ARIA indicate that (i) disease control is being considered for future initiatives [49] and (ii) methods for measuring severity and control in allergic disease must be uniform [49]. The adoption of control-based approaches in AR is likely to modify the physician-patient relationship. The CARAT, RCAT and ARCT are multi-item questionnaires that require the patient to provide a fair amount of information on his/her recent condition. However, since control is largely a patient-led concept, remote measurements (by 'phone or over the Internet) could conceivably reduce the frequency of face-to-face consultations. Nevertheless, as noted by Glasziou et al. [50], the benefits of monitoring the response to treatment, detecting adverse effects and gauging the need to adjust treatment must be balanced against inconvenience, cost and the potential impact of false positives and false negatives of disease control. Measurements of control must therefore be reproducible, quick and easy to perform in routine practice and should focus on the disease's impact in everyday life. For example, one could consider an approach in which a patient's degree of disease control is simply equated to the "strength" of the medication (i.e. therapeutic pressure) that he/she has to take in order to gain sufficient relief from his/her symptoms.

## Conclusion

While the ARIA classification of the severity of AR is useful, it is not an optimal guide for making everyday patient management decisions, especially in patients already on therapy. Experience in asthma suggests that there are good reasons to consider measuring control on a routine basis in AR, as a complement to ARIA's severity-based approach. There is a need to compare existing tools and perhaps develop new ones. Regardless of the details of control-based classifications in AR, the key challenge for any instrument will be to achieve high levels of physician awareness, uptake and application - which should ultimately lead to better patient outcomes.

## Abbreviations

ACS: Allergy-Control-SCORE; AR: Allergic rhinitis; ARC: Allergic Rhinitis Control; ARCT: Allergic Rhinitis Control Test; ARIA: Allergic Rhinitis and its Impact on Asthma; CARAT: Control of Allergic Rhinitis and Asthma Test; ENT: Ear, nose and throat; GINA: Global Initiative for Asthma; H1A: H1-antihistamine; ICS: Intranasal corticosteroid; PCP: Primary care physician; RCAT: Rhinitis Control Assessment Test; RQLQ: Rhinitis Quality of Life Questionnaire; VAS: Visual analogue scale.

## Competing interests

Pascal Demoly is a consultant and a speaker for Stallergenes, ALK and Chiesi and was a speaker for Merck, Astra Zeneca, Menarini and GlaxoSmithKline in 2010–2012. Moises Calderon has received consulting fees, honoraria for lectures and/or research funding from ALK-Abello, Merck, Stallergenes and Allergopharma. Glenis Scadding has received consulting fees, honoraria for lectures and/or research funding from ALK-Abello, GSK, Meda, Merck and Stallergenes. Thomas Casale has received consulting fees from Stallergenes and has been an investigator on grants awarded to Creighton University by Stallergenes and Schering-Plough-MSD. This work was supported by an educational grant from Stallergenes.

## Authors’ contributions

PD, MC, TC, GS, IAM, JJB, BD, TH, OM, FT and ES all made substantial contributions to the (i) the conception and design of the disease control classification and (ii) the identification and review of relevant literature on ARIA and published proposals for modification. Likewise, PD, MC, TC, GS, IAM, JJB, BD, TH, OM, FT and ES were all involved in drafting the manuscript and/or revising it critically for important intellectual content. All the authors have given final approval of the version to be published.

## References

[B1] Katelaris CH Lee BW Potter PC Maspero JF Cingi C Lopatin A Saffer M Xu G Walters RD Prevalence and diversity of allergic rhinitis in regions of the world beyond Europe and North America Clin Exp Allergy 2012 42 186 207 10.1111/j.1365-2222.2011.03891.x 22092947

[B2] Bauchau V Durham SR Epidemiological characterization of the intermittent and persistent types of allergic rhinitis Allergy 2005 60 350 353 10.1111/j.1398-9995.2005.00751.x 15679721

[B3] Asher MI Montefort S Björkstén B Lai CK Strachan DP Weiland SK Williams H ISAAC Phase Three Study Group Worldwide time trends in the prevalence of symptoms of asthma, allergic rhinoconjunctivitis, and eczema in childhood: ISAAC Phases One and Three repeat multicountry cross-sectional surveys Lancet 2006 368 733 743 10.1016/S0140-6736(06)69283-0 16935684

[B4] Nathan RA The burden of allergic rhinitis Allergy Asthma Proc 2007 28 3 9 10.2500/aap.2007.28.2934 17390749

[B5] Léger D Annesi-Maesano I Carat F Rugina M Chanal I Pribil C El Hasnaoui A Bousquet J Allergic rhinitis and its consequences on quality of sleep. An unexplored area Arch Intern Med 2006 166 1744 1748 10.1001/archinte.166.16.1744 16983053

[B6] Meltzer EO Quality of life in adults and children with allergic rhinitis J Allergy Clin Immunol 2001 108 S45 53 10.1067/mai.2001.115566 11449206

[B7] Meltzer EO Bukstein DA The economic impact of allergic rhinitis and current guidelines for treatment Ann Allergy Asthma Immunol 2011 106 S12 16 10.1016/j.anai.2010.10.014 21277528

[B8] Shaaban R Zureik M Soussan D Neukirch C Heinrich J Sunyer J Wjst M Cerveri I Pin I Bousquet J Jarvis D Burney PG Neukirch F Leynaert B Rhinitis and onset of asthma: a longitudinal population-based study Lancet 2008 372 1049 1057 10.1016/S0140-6736(08)61446-4 18805333

[B9] Valovirta E Myrseth SE Palkonen S The voice of the patients: allergic rhinitis is not a trivial disease Curr Opin Allergy Clin Immunol 2008 8 1 9 10.1097/ACI.0b013e3282f3f42f 18188010

[B10] Canonica GW Bousquet J Mullol J Scadding GK Virchow JC A survey of the burden of allergic rhinitis in Europe Allergy 2007 62 Suppl 85 17 25 17927674 10.1111/j.1398-9995.2007.01549.x

[B11] Marple BF Fornadley JA Patel AA Fineman SM Fromer L Krouse JH Lanier BQ Penna P American Academy of Otolaryngic Allergy Working Group on Allergic Rhinitis Keys to successful management of patients with allergic rhinitis: focus on patient confidence, compliance, and satisfaction Otolaryngol Head Neck Surg 2007 136 S107 124 10.1016/j.otohns.2007.02.031 17512862

[B12] Wallace DV Dykewicz MS Bernstein DI Blessing-Moore J Cox L Khan DA Joint Task Force on Practice, American Academy of Allergy, Asthma & Immunology, American College of Allergy, Asthma and Immunology, Joint Council of Allergy, Asthma and Immunology The diagnosis and management of rhinitis: an updated practice parameter J Allergy Clin Immunol 2008 122 2 Suppl S1 84 18662584 10.1016/j.jaci.2008.06.003

[B13] Bousquet J van CP Khaltaev N ARIA Workshop Group, World Health Organization Allergic rhinitis and its impact on asthma J Allergy Clin Immunol 2001 108 S147 334 10.1067/mai.2001.118891 11707753

[B14] Bousquet J Khaltaev N Cruz AA Denburg J Fokkens WJ Togias A Zuberbier T Baena-Cagnani CE Canonica GW van Weel C Agache I Aït-Khaled N Bachert C Blaiss MS Bonini S Boulet LP Bousquet PJ Camargos P Carlsen KH Chen Y Custovic A Dahl R Demoly P Douagui H Durham SR van Wijk RG Kalayci O Kaliner MA Kim YY Kowalski ML Allergic Rhinitis and its Impact on Asthma (ARIA) 2008 Update (in collaboration with the World Health Organization, GA2LEN and AllerGen) Allergy 2008 63 S8 160 10.1111/j.1398-9995.2007.01620.x 18331513

[B15] Brozek JL Bousquet J Baena-Cagnani CE Bonini S Canonica GW Casale TB van Wijk RG Ohta K Zuberbier T Schünemann HJ Global Allergy and Asthma European Network, Grading of Recommendations Assessment, Development and Evaluation Working Group Allergic Rhinitis and its Impact on Asthma (ARIA) guidelines: 2010 revision J Allergy Clin Immunol 2010 126 466 476 10.1016/j.jaci.2010.06.047 20816182

[B16] Demoly P Allaert FA Lecasble M Bousquet J Validation of the classification of ARIA (allergic rhinitis and its impact on asthma) Allergy 2003 58 672 675 10.1034/j.1398-9995.2003.t01-1-00202.x 12823130

[B17] Demoly P Concas V Urbinelli R Allaert FA Spreading and impact of the World Health Organization's Allergic Rhinitis and its impact on asthma guidelines in everyday medical practice in France. Ernani survey Clin Exp Allergy 2008 38 1803 1807 18721255 10.1111/j.1365-2222.2008.03085.x

[B18] Global Strategy for Asthma Management and Prevention Global Initiative for Asthma (GINA) 2011 http://www.ginasthma.org

[B19] WHO Collaborating Center for Asthma and Rhinitis Severe chronic allergic (and Related) diseases: a uniform approach - A MeDALL - GA (2)LEN - ARIA position paper Int Arch Allergy Immunol 2012 158 216 231 22382913 10.1159/000332924

[B20] del Cuvillo A Montoro J Bartra J Valero A Ferrer M Jauregui I Dávila I Sastre J Mullol J Validation of ARIA duration and severity classifications in Spanish allergic rhinitis patients - The ADRIAL cohort study Rhinology 2010 48 201 205 20502761 10.4193/Rhin09.099

[B21] Jáuregui I Dávila I Sastre J Bartra J del Cuvillo A Ferrer M Montoro J Mullol J Molina X Valero A Validation of ARIA (Allergic Rhinitis and its Impact on Asthma) classification in a pediatric population: the PEDRIAL study Pediatr Allergy Immunol 2011 22 388 392 10.1111/j.1399-3038.2010.01108.x 21261745

[B22] Bousquet J Neukirch F Bousquet PJ Gehano P Klossek JM Le Gal M Allaf B Severity and impairment of allergic rhinitis in patients consulting in primary care J Allergy Clin Immunol 2006 117 158 162 10.1016/j.jaci.2005.09.047 16387600

[B23] Ramirez LF Urbinelli R Allaert FA Demoly P Combining H1-antihistamines and nasal corticosteroids to treat allergic rhinitis in general practice Allergy 2011 66 1501 1502 10.1111/j.1398-9995.2011.02682.x 21883275

[B24] Van Hoecke H Vastesaeger N Dewulf L De Bacquer D van Cauwenberge P Is the allergic rhinitis and its impact on asthma classification useful in daily primary care practice? J Allergy Clin Immunol 2006 118 758 759 10.1016/j.jaci.2006.05.015 16950299

[B25] Bousquet PJ Bousquet-Rouanet L Co Minh HB Urbinelli R Allaert FA Demoly P ARIA (Allergic Rhinitis and Its Impact on Asthma) classification of allergic rhinitis severity in clinical practice in France Int Arch Allergy Immunol 2007 143 163 169 10.1159/000099307 17284924

[B26] Bousquet J Bachert C Canonica GW Casale TB Cruz AA Lockey RJ Zuberbier T Extended Global Allergy and Asthma European Network, World Allergy Organization and Allergic Rhinitis and its Impact on Asthma Study Group Unmet needs in severe chronic upper airway disease (SCUAD) J Allergy Clin Immunol 2009 124 428 433 10.1016/j.jaci.2009.06.027 19660803

[B27] Valero A Ferrer M Sastre J Navarro AM Monclús L Martí-Guadaño E Herdman M Dávila I Del Cuvillo A Colás C Baró E Antépara I Alonso J Mullol J A new criterion by which to discriminate between patients with moderate allergic rhinitis and patients with severe allergic rhinitis based on the Allergic Rhinitis and its Impact on Asthma severity items J Allergy Clin Immunol 2007 120 359 365 10.1016/j.jaci.2007.04.006 17531304

[B28] Valero A Munoz-Cano R Sastre J Navarro AM Marti-Guadano E Davila I Del Cuvillo A Colas C Antepara I Izquierdo I Mullol J The impact of allergic rhinitis on symptoms, and quality of life using the new criterion of ARIA severity Rhinology 2012 50 33 36 22469603 10.4193/Rhino.11.071

[B29] Demoly P Urbinelli R Allaert FA Bousquet PJ Should we modify the allergic rhinitis and its impact on asthma dichotomic classification of severity? Allergy 2010 65 1488 1490 10.1111/j.1398-9995.2010.02374.x 20415715

[B30] Bousquet PJ Combescure C Neukirch F Klossek JM Mechin H Daures JP Bousquet J Visual analog scales can assess the severity of rhinitis graded according to ARIA guidelines Allergy 2007 62 367 372 10.1111/j.1398-9995.2006.01276.x 17362246

[B31] Spector SL Nicklas RA Chapman JA Bernstein IL Berger WE Blessing-Moore J Dykewicz MS Fineman SM Lee RE Li JT Portnoy JM Schuller DE Lang D Tilles SA Joint Task Force on Practice Parameters, American Academy of Allergy, Asthma, and Immunology, American College of Allergy, Asthma, and Immunology, Joint Council of Allergy, Asthma, and Immunology Symptom severity assessment of allergic rhinitis: part 1 Ann Allergy Asthma Immunol 2003 91 105 114 10.1016/S1081-1206(10)62160-6 12952100

[B32] Rouve S Didier A Demoly P Jankowsky R Klossek JM Annesi-Maesano I Numeric score and visual analog scale in assessing seasonal allergic rhinitis severity Rhinology 2010 48 285 291 21038018 10.4193/Rhino09.208

[B33] Farnik M Pierzchała W Instrument development and evaluation for patient-related outcomes assessments Patient Related Outcome Measures 2012 3 1 7 22915979 10.2147/PROM.S14405 PMC3417934

[B34] Nogueira-Silva L Martins SV Cruz-Correia R Azevedo LF Morais-Almeida M Bugalho-Almeida A Vaz M Costa-Pereira A Fonseca JA Control of allergic rhinitis and asthma test–a formal approach to the development of a measuring tool Respir Res 2009 10 52 10.1186/1465-9921-10-52 19534774 PMC2706215

[B35] Fonseca JA Nogueira-Silva L Morais-Almeida M Azevedo L Sa-Sousa A Branco-Ferreira M Fernandes L Bousquet J Validation of a questionnaire (CARAT10) to assess rhinitis and asthma in patients with asthma Allergy 2010 65 1042 1048 10.1111/j.1398-9995.2009.02310.x 20121755

[B36] Fonseca JA Nogueira-Silva L Morais-Almeida M Sa-Sousa A Azevedo LF Ferreira J Branco-Ferreira M Rodrigues-Alves R Bugalho-Almeida A Bousquet J Control of Allergic Rhinitis and Asthma Test (CARAT) can be used to assess individual patients over time Clin Transl Allergy 2012 2 16 10.1186/2045-7022-2-16 22935298 PMC3520832

[B37] Juniper EF O'Byrne PM Guyatt GH Ferrie PJ King DR Development and validation of a questionnaire to measure asthma control Eur Respir J 1999 14 902 7 10.1034/j.1399-3003.1999.14d29.x 10573240

[B38] Nathan RA Dalal AA Stanford RH Meltzer EO Schatz M Derebery J Mintz M Thompson MA Dibenedetti DB Qualitative Development of the Rhinitis Control Assessment Test (RCAT), an Instrument for Evaluating Rhinitis Symptom Control Patient 2010 3 91 99 10.2165/11318410-000000000-00000 22273360

[B39] Schatz M Meltzer EO Nathan R Derebery MJ Mintz M Stanford RH Dalal AA Silvey MJ Kosinski M Psychometric validation of the rhinitis control assessment test: a brief patient-completed instrument for evaluating rhinitis symptom control Ann Allergy Asthma Immunol 2010 104 118 124 10.1016/j.anai.2009.11.063 20306814

[B40] Demoly P Jankowski R Chassany O Bessah Y Allaert FA Validation of a self-questionnaire for assessing the control of allergic rhinitis Clin Exp Allergy 2011 41 860 868 10.1111/j.1365-2222.2011.03734.x 21518040

[B41] Nathan RA Sorkness CA Kosinski M Schatz M Li JT Marcus P Murray JJ Pendergraft TB Development of the asthma control test: a survey for assessing asthma control J Allergy Clin Immunol 2004 113 59 65 10.1016/j.jaci.2003.09.008 14713908

[B42] Häfner D Reich K Matricardi PM Meyer H Kettner J Narkus A Prospective validation of 'Allergy-Control-SCORE(TM)': a novel symptom-medication score for clinical trials Allergy 2011 66 629 636 10.1111/j.1398-9995.2010.02531.x 21261656

[B43] Calderon MA Eichel A Makatsori M Pfaar O Comparability of subcutaneous and sublingual immunotherapy outcomes in allergic rhinitis clinical trials Curr Opin Allergy Clin Immunol 2012 12 249 256 10.1097/ACI.0b013e32835358b3 22499145

[B44] Scadding G Batten TN Skinner MA Dunham KA Capella A Calderon M Identification of the Cut-off Point Between Adequately and Inadequately Controlled rhinoconjunctivitis/rhinitis using the mini-Rhinoconjunctivitis Quality Of Life Questionnaire 2012 Nottingham, UK: Poster presentation at the The British Society for Allergy & Clinical Immunology Annual Conference

[B45] Humbert M Holgate S Boulet LP Bousquet J Asthma control or severity: that is the question Allergy 2007 62 95 101 17298415 10.1111/j.1398-9995.2006.01308.x

[B46] Braido F Bousquet PJ Brzoza Z Canonica GW Compalati E Fiocchi A Fokkens W Gerth Van Wijk R La Grutta S Lombardi C Maurer M Pinto AM Ridolo E Senna GE Terreehorst I Todo Bom A Bousquet J Zuberbier T Baiardini I Specific recommendations for PROs and HRQoL assessment in allergic rhinitis and/or asthma: a GA(2)LEN taskforce position paper Allergy 2010 65 959 968 10.1111/j.1398-9995.2010.02383.x 20486919

[B47] Juniper EF O'Byrne PM Ferrie PJ King DR Roberts JN Measuring asthma control. Clinic questionnaire or daily diary Am J Respir Crit Care Med 2000 162 1330 1334 11029340 10.1164/ajrccm.162.4.9912138

[B48] Liu AH Zeiger R Sorkness C Mahr T Ostrom N Burgess S Rosenzweig JC Manjunath R Development and cross-sectional validation of the Childhood Asthma Control Test J Allergy Clin Immunol 2007 119 817 825 10.1016/j.jaci.2006.12.662 17353040

[B49] Bousquet J World Health Organization Collaborating Center for Asthma and Rhinitis Allergic Rhinitis and its Impact on Asthma (ARIA): Achievements in 10 years and future needs J Allergy Clin Immunol 2012 130 1049 62 10.1016/j.jaci.2012.07.053 23040884

[B50] Glasziou P Irwig L Mant D Monitoring in chronic disease: a rational approach BMJ 2005 330 644 648 10.1136/bmj.330.7492.644 15774996 PMC554914

